# Prolactin as immune cell regulator in *Toxocara canis* somatic larvae chronic infection

**DOI:** 10.1042/BSR20180305

**Published:** 2018-07-31

**Authors:** Víctor Hugo Del Río-Araiza, Karen Elizabeth Nava-Castro, Fernando Alba-Hurtado, Andrés Quintanar-Stephano, Hugo Aguilar-Díaz, Marco Antonio Muñoz-Guzmán, Pedro Ostoa-Saloma, María Dolores Ponce-Regalado, Jorge Morales-Montor

**Affiliations:** 1 Departamento de Inmunología, Instituto de Investigaciones Biomédicas, Universidad Nacional Autónoma de México, AP 70228, México D.F. 04510, México; 2 Departamento de Genotoxicología, Centro de Ciencias de la Atmósfera, Universidad Nacional Autónoma de Mèxico; 3 Departamento de Ciencias Biológicas, Facultad de Estudios Superiores Cuautitlán, Universidad Nacional Autónoma de México, México; 4 Centro de Ciencias Básicas, Universidad Autónoma de Aguascalientes, Aguascalientes, México; 5 Centro Nacional de Investigación Disciplinaria en Parasitología Veterinaria, Instituto Nacional de Investigaciones Forestales Agrícolas y Pecuarias, INIFAP, Jiutepec, Morelos, México; 6 Universidad de Guadalajara, Centro Universitario de los Altos - Departamento de clínicas. Carretera a Yahualica, Km. 7.5. Tepatitlán de Morelos, Jalisco, México

**Keywords:** Chronic infection, Host-Parasite Interaction, immunomodulation, immune response, Prolactin, Toxocara cannis

## Abstract

Toxocariasis is a zoonotic disease produced by ingestion of larval *Toxocara* spp. eggs. Prolactin (PRL) has been considered to have an important role in *Toxocara canis* infection. Recent evidence has found that PRL directly can increase parasite growth and differentiation of *T. canis*. The present study, evaluated the effect of high PRL levels on the immune system’s response and parasites clearance in chronic infection. Our results showed that hyperprolactinemia did not affect the number of larvae recovered from several tissues in rats. Parasite-specific antibody production, showed no difference between the groups. Lung tissue presented eosinophilic granulomas typical of a chronic infection in all the experimental groups. Flow cytometry analysis was made in order to determine changes in the percentage of innate and adaptive immune cell subpopulations in the spleen, peripheric (PLN) and mesenteric (MLN) lymphatic nodes. The results showed a differential effect of PRL and infection on different immune compartments in the percent of total T cells, T helper cells, T cytotoxic cells, B cells, NK cells, and Tγδ cells. To our knowledge, for the first time it is demonstrated that PRL can have an immunomodulatory role during *T. canis* chronic infection in the murine host.

## Introduction

*Toxocara canis* (*T. canis*) is the main helminth responsible for Toxocariasis, a disease of medical and veterinarian importance. Adult worms of this parasite usually have dogs, foxes, coyotes, and wolves (mainly pups) as definitive hosts. In Mexico, and in many other places, it is the most commonly found helminth in dogs [1]. *T. canis* also has paratenic hosts, amongst which can be found most mammals (e.g. humans, pigs, sheep, rats, mice etc.), birds and invertebrates such as earthworms and arthropods such as fleas. Toxocariasis is considered as one of the most widely distributed zoonoses due to the ample and close relationship between humans and domestic cats and dogs [2]. The biological cycle of *T. canis* is complex and varies depending on the host type (definitive or paratenic), host’s age (pup or adult), and physiological state (gestating or non-gestating). In pregnant bitches, somatic larvae go through a reactivation process consisting of the migration of larvae toward the uterus and mammary glands. It has been previously reported that this process is started by an increased concentration of Prolactin (PRL) in the bloodstream [3]. According to literature review, there are few studies on the role of PRL on *T. canis* infection; however, a study performed in a murine model suggests that the reactivation and migration of somatic larvae toward the uterus and mammary glands could be started by an increased level of this hormone, thus favoring transplacental and lactogenic transmission into the offspring [4]. Another study performed by Reiterová et al. [5] with the murine model reported the presence of *T. canis* larvae in mice pups 5 days post-birth, thus evidencing the role of lactogenic transmission in this parasite’s life cycle. Besides the apparent role of PRL on *T. canis* larvae reactivation, this hormone also has an immunomodulatory role that has been demonstrated in several studies; Nagy and Berkzi [6] proved that PRL, growth hormone, and placental lactogen administration in previously hypophysectomized rats, that were undergoing an immunodeficient course, had their immune activity restored after the treatment. Another experiment utilizing bromocriptine (a dopaminergic agonist) to selectively inhibit PRL secretion showed similar results, meaning that the decreased immune response, both humoral and cellular, is restored after bromocriptine withdrawal [7]. Moreover, it has also been reported that the immune system is capable of regulating PRL secretion. Cytokines interleukin (IL)-1, IL-6, and TNF-α can also act as endocrine regulators of hypophysis PRL secretion [8]. Therefore, PRL is currently considered not only as a hormone but also as a cytokine possessing distinct immunomodulating qualities.

Concerning the immune response, it has been reported that during larvae migration inside the host, an adaptive type response is triggered targetting excretion and secretion antigens (TES-Ag) of the parasite, this response is characterized by an increased number of T lymphocytes (TL), helper TLs (Th), and cytotoxic TLs (CTLs). On the other hand, it also stimulates macrophages to produce ILs. IL-4 promotes a Th2 response increasing cytokines such as IL-5, IL-6, and IL-13 [9–11]. An increased IL-4 stimulates in turn the proliferation and maturation of B lymphocytes (BL), isotype IgM switch to IgE and specific IgG production, besides contributing to the stimulation of mastocytes that elevate the inflammatory response. These antigens also induce an increased concentration of IL-5, a powerful eosinophil inductor [12], characteristic of this infection. In addition to stimulating a Th2 response, an increased plasmatic level of IFN-γ has been observed in murine models, characteristic of a Th1 response [13]. IFN-γ acts on T, BL, NK cells, and macrophages; it is a key modulator of cell-mediated immunity. An association between IFN-γ and IL-3 has been described in the formation of eosinophilic granulomas in pathogen-related diseases (schistosomiasis) or in autoimmune diseases. These granulomas are also present due to *T. canis* chronic infection in the different organs and tissues through which it migrates [14,15]. Often, larvae remain within these granulomas until stimulated to do otherwise, by PRL per example, thus triggering their migration toward the uterus and mammary glands in order to be transmitted into the offspring.

Based on these observations, the present study aims to evaluate the immune system’s behavior and the migration of *T. canis* somatic larvae under normal and hyperprolactinemia conditions in a murine model, seeking a better understanding of a host-generated response and the possible transregulating mechanisms of the parasite.

## Materials and methods

### Ethics statement

Animal care and experimentation practices at Universidad de Aguascalientes and the Instituto de Investigaciones Biomédicas were constantly evaluated and approved by both Institute’s Animal Care and Use Committee (Comité de Cuidado y Uso de Animales de Experimentación, CICUAL, permit number: 201-2016) adhering to the official Mexican regulations (NOM-062-ZOO-1999). Mexican regulations are in strict accordance with the recommendations in the Guide for the Care and Use of Laboratory Animals of the National Institutes of Health (NIH) of the U.S.A., to ensure compliance with established international regulations and guidelines. The rats were killed using anesthesia overdose (Sevorane®) followed by decapitation. Efforts were made to minimize suffering.

### Animals

A total of 30 Wistar male rats were used (2 months old) in each round of experiments, kindly provided by the Bioterium of the Instituto de Investigaciones Biomédicas (IIB), Universidad Nacional Autónoma de Mexico (UNAM). The animals were organized into six groups and allocated in polycarbonate boxes (50 cm l × 23 cm w × 21 cm h). The groups were distributed as follows: (i) untreated control (Int-Ctrl *n*=5), (ii) untreated infected (Int-Infx *n*=5), (iii) simulated adenohypophysis implant in renal capsule surgery (Sham) control (Sh-HPRL-Ctrl *n*=5), (iv) simulated adenohypophysis implant in renal capsule surgery (Sham) infected (Sh-HPRL-Infx *n*=5), (v) adenohypophysis implant in renal capsule surgery control (HPRL-Ctrl *n*=5), and (vi) adenohypophysis implant in renal capsule surgery infected (HPRL-Infx *n*=5). Two rounds of experiments were performed. Animals were kept in cycles of 12 h of light/darkness. Water and food (Harlan 2019S Teklad Global 19% Protein Extruded Rodent Diet Sterilizable) were supplied *ad libitum* in sterile conditions.

### Harvesting and processing of *T. canis* eggs

*T. canis* eggs were obtained from adult parasites collected from the small intestine of naturally infected pups, killed humanely at the Centro de Control Canino de Cuautitlán, Estado de Mexico. Females were separated, rinsed in tap water, and placed in 1× PBS. Afterward, uteri were collected by incising the first third of the body. Uteri were placed in physiological saline solution (PSS), eggs were obtained using a fine pore filter. The ova were washed several times in 1× PBS and centrifuged at 3250 ***g*** for 5 min. The sedimented eggs were resuspended in a 1× PBS/2% formaldehyde solution and incubated at 27°C for 28 days to obtain the infective form (embryonated eggs (EE)).

### *T. canis* infection in rats

Previous to infection, the inoculum was washed three times to eliminate the PBS/formaldehyde solution. Eggs were then resuspended in PSS and concentrated to 2000 EE per ml. Infection was performed by intragastric administration using a type Foley metallic probe at 8 weeks of age inoculating 2000 EE per rat.

### Surgical procedures and culling

The procedures were performed at 45 days post-infection, the performed surgeries were: simulated adenohypophysis implant in renal capsule (Sham) and adenohypophysis implant in renal capsule. The adenohypophysis for the implants were obtained from littermates donating females. A total of four adenohypophysis were implanted per rat. To perform the surgery, the back of the rat is shaved to the level of the kidneys and placed on the surgery table in the prone position. The area is cleaned with benzalkonium chloride and an incision is made 2–3 cm above the skin media line. Subsequently, the right kidney is exposed dorsally and with the insulin needle a small incision is made in the renal capsule. Using thin transplants, a sac is formed between the capsule and the kidney being very careful not to break it. When the sac is ready, the adenohypophysis is placed deep inside it, the kidney is returned to its original position and the dorsal wound is sutured. The average time between the pituitary excision and completion of the transplantation was 4–6 min. In general, total surgical time did not exceed 15 min, and full recovery of the animals occurred within 20–30 min [16]. At the end of surgery, the rats were placed in a recuperation chamber (clinical O_2_ + thermic mattress) and administered 5000 UI procaine penicillin G IM every 24 h for 3 days. The animal culling was performed 25 days after surgery by anesthesia overdose (Sevorane®) followed by decapitation. Immediately after euthanasia, several tissues were collected, i.e. blood, spleen, liver, lungs, brain, right kidney, peripheral lymph nodes (PLN) and mesenteric lymph nodes (MLN). Concerning the infected animals, the mammary gland was collected by scraping the subcutaneous tissue in the corresponding area.

### PRL levels in serum

PRL levels titration in serum was evaluated using the commercial kit PRL rat ELISA (ALPCO®) following the manufacturer’s instructions. Results were expressed in ng/ml.

### Larvae recovery

After the moment of euthanasia, lungs, brain, and mammary glands of infected animals were weighted and macerated; tissue was digested in artificial gastric juice (1% pepsin (250 units/mg, SIGMA®) and 1% HCl 37% (pH: 2.0) (10 ml of artificial gastric juice/ 1 g of tissue) for 24 h. Samples were centrifuged at 791 ***g*** for 5 min, and the pellet was resuspended in 1 ml 4% paraformaldehyde. The parasite count involved ten counts per 20 μl sample, the total number of larvae was multiplied 50 times to calculate the number of larvae per milliliter and therefore the number of larvae per gram of tissue, this number was then multiplied times the total weight of the collected organ resulting in the total number of larvae.

### Histopathology

Tissue samples (4 × 4 × 4 mm = 0.6 mm^3^) were taken from lung, liver, and kidney. Histological sections of 4 µm thickness were made using low profile blades in the microtome, the sections were mounted in slides previously treated with acid alcohol and a 1:10 poly-l-lysine solution; sections were stained with Hematoxylin/Eosin and observed under the microscope at 20× amplification.

### Flow cytometry

Spleen, PLN and MLN collected at the time of killing were disaggregated using a sterile nylon mesh (70 μm) and a syringe plunge in 1× PBS pH 7.4/4°C. The cell suspension was centrifuged at 182 ***g*** for 3 min, decanted and resuspended in in 500 μl of erythrocyte lysis buffer (spleen only), incubated for 10 min at room temperature; 700 μl of FACS buffer were added followed by centrifugation at 182 ***g*** for 3 min. The supernatant was decanted and the cells resuspended in 500 μl of FACS buffer from which 25 μl were taken and the cells fixed with 4% paraformaldehyde for 10 min/37°C in a 96-well plate. Cells were centrifuged at 127 ***g*** for 5 min and washed with 200 μl FACS buffer; 150 μl absolute methanol were added and incubated for 10 min/4°C. The plate was centrifuged again at 506 ***g*** for 3 min, and washed with 200 μl FACS buffer; 150 μl of primary antibody solution were added to the corresponding wells and incubated for 10 min/4°C. After incubation (following primary antibodies were used: AF 488 anti-rat CD3 (Biolegend, Clone 1F4), PE-Cy5 mouse anti-rat CD4 (BD Biosciences, Clone Ox-35), PE Mouse anti-rat CD8α (BD Biosciences, Clone Ox-8), PE anti-rat CD45RA (Biolegend, Clone Ox-33), PE anti-rat TCRγδ (Biolegend, Clone V65), and AF647 anti-rat CD161 (Biolegend, Clone 1F4)). Wells were washed with 150 μl FACS buffer after incubation. Cells were finally resuspended in 200 μl of FACS buffer and stored at 4°C in the dark. Data analysis was performed using the software FlowJo v7.6.

### TES-Ag purification

TES-Ag was obtained by culturing the *T. canis* larvae according to the method described by de Savigny [17] Purity and integrity of ESA was determined by SDS/PAGE and Coomassie staining, protein content was quantitated by Bradford method [18].

### *T. canis* specific IgG determination

The levels of ESA targetting antibodies in serum were measured indirectly by ELISA optimized according to the antigen’s concentration, serum, and conjugate’s dilution. Antigen concentration was 1 µg/ml, serum dilution was of 1:200 and the conjugate (rat IgG) was diluted as 1:10000. Plate reading was done at 492 nm, 15 s agitation in an ELISA plate reader (Multiscan Ascent).

### Statistical analysis

Data were showed as the mean average ± S.D. The results obtained are shown in bar graphics, describing the mean average and S.D. These values were evaluated by two-way ANOVA and Bonferroni multiple comparison amongst all groups. A significant difference was considered when *P*<0.05; the software GraphPad Prism v6.0 was used.

## Results

### PRL levels in serum

**Figure 1:**
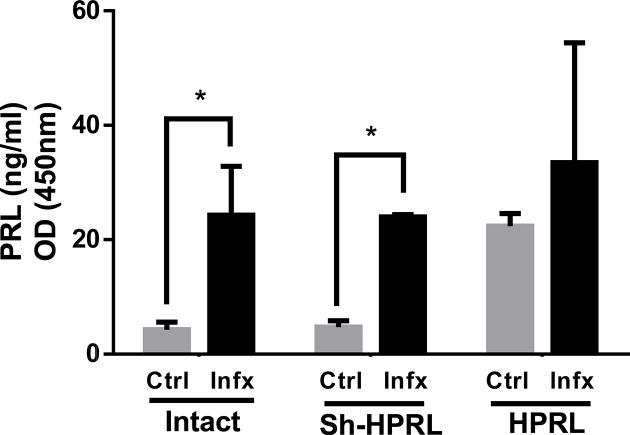
Means (± S.D.) of PRL levels in serum from experimental rats We used five animals per group, two rounds of experiments. Infection was performed by intragastric administration using a type Foley metallic probe at 8 weeks of age inoculating 2000 *T. canis* eggs per rat. PRL level in serum was evaluated using an ELISA commercial kit. Results were expressed in ng/ml. Data are shown as the mean average ± S.D. These values were evaluated by two-way ANOVA and Bonferroni multiple comparisons amongst all groups. A significant difference was considered when *P*<0.05. Abbreviations: Ctrl; uninfected rats, HPRL; adenohypophysis implant in renal capsule surgery; Infx; infected rats, Intact; untreated rats, Sh-HPRL; simulated adenohypophysis implant in renal capsule surgery.*P<0.05.

Figure 1 shows the concentration level of PRL obtained in the Int-Ctrl, Int-Infx, Sh-HPRL-Ctrl, Sh-HPRL-Infx, HPRL-Ctrl, and HPRL-Infx groups. An increased PRL concentration can be observed due to infection in the Intact and Sh-HPRL groups compared with the control (*P*<0.05). Also, note that in the figure that PRL levels are also significantly increased in the HPRL-Ctrl group as compared with the non-infected Int-Ctrl and Sh-HPRL-Ctrl groups.

### Larval counts

**Figure 2:**
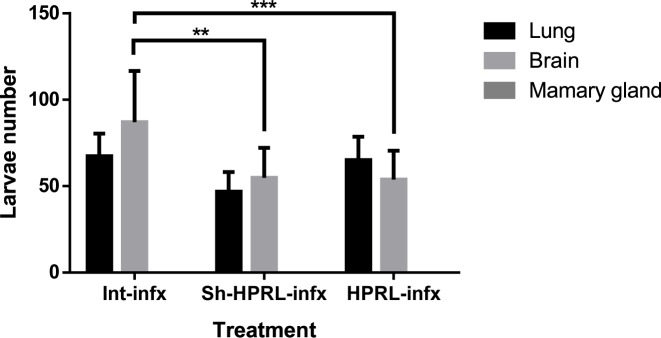
Mean (± S.D.) of recuperated larvae number through the digestion of lungs and brain of *T. canis* infected male rats with different treatments We used five animals per group, two rounds of experiments. Infection was performed by intragastric administration using a type Foley metallic probe at 8 weeks of age inoculating 2000 *T. canis* eggs per rat. After of the moment of euthanasia, lungs, brain, and mammary glands of infected animals were digested in artificial gastric juice for 24 h. No larvae were recuperated from mammary glands. Data were shown as the mean average ± S.D. These values were evaluated by two-way ANOVA and Bonferroni multiple comparison a all groups. A significant difference was considered when *P*<0.05. **P<0.01; ***P<0.001. Abbreviations: Infx; infected rats, Intact; untreated rats, HPRL; adenohypophysis implant in renal capsule surgery; Sh-HPRL; simulated adenohypophysis implant in renal capsule surgery.

Parasitic load of lung, brain, and mammary gland is shown in Figure 2. No larvae were recovered from mammary glands in any of the experimental groups. The larvae number recovery in lungs, at 70 days post-infection, were in groups: (i) Int: 67.32 ± 13.15, (iv) Sh-HPRL: 46.8 ± 11.34, and (v) HPRL: 65 ± 13.62. In brain, percentages were: (i) Int: 87 ± 29.74, (iv) Sh-HPRL: 54.83 ± 17.37, and (v) HPRL: 53.76 ± 16.8. We found that a significant decrease in larvae number was observed in the brains of Sh-HPRL and HPRL rat groups.

### Histopathology

**Figure 3:**
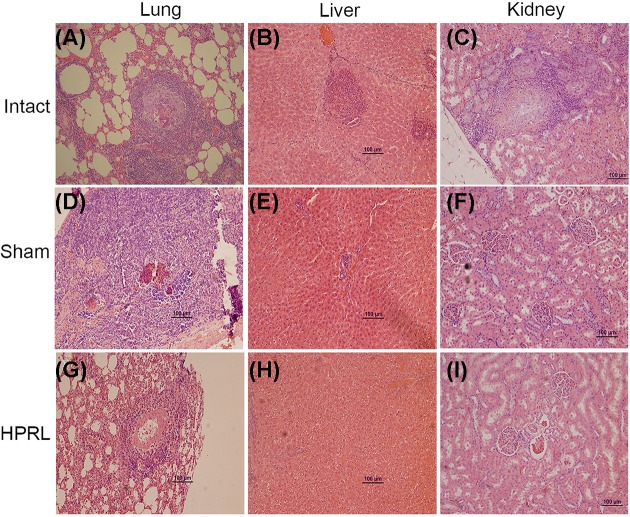
Histologic sections of lung, liver and kidney from infected rats. (**A**) Lung intact. (**B**) Liver intact. (**C**) Kidney intact. (**D**) Lung Sham. (**E**) Liver Sham. (**F**) Kidney Sham, (**G**) Lung HPRL. (**H**) Liver HPRL. (**I**) Kidney HPRL; 20×. Characteristic eosinophilic granulomas can be observed with abundant infiltrate inflammation. The exterior zone is mainly composed of fibrocytes and collagen. In the interior zone, eosinophils and larvae can be observed. We used five animals per group and two rounds of experiments. Infection was performed by intragastric administration using a type Foley metallic probe at 8 weeks of age inoculating 2000 *T. canis* eggs per rat. Tissue samples were taken from lung, liver, and kidney. Histological sections were made using low profile blades in the microtome, the sections were mounted in slides previously treated with acid alcohol and a 1:10 poly-l-lysine solution; sections were stained with Hematoxylin/Eosin and observed under the microscope at 20× amplification. Abbreviations: Intact; untreated rats, HPRL; adenohypophysis implant in renal capsule surgery; Sham; simulated adenohypophysis implant in renal capsule surgery.

In order to evaluate the effect of PRL on the reactivation of *T. canis* larvae, the histological differences were examined concerning the inflammatory response to infiltration in lungs, liver, and kidney between the infected experimental groups (Figure 3).

In lungs (Figure 3A,D,G), the presence of granulomas, collagen compounds, and a core containing epithelioid cells, giant multinucleated cells, and macrophages, as well as the presence of larvae or remains of the same can be observed. Larvae are often found within a blanket of eosinophils. Outside the collagen capsule of each larva containing granuloma, the presence of a mixed infiltrate was found, predominantly of lymphocytic with a moderate number of eosinophils and neutrophils. Larva-exempted granulomas also had a collagen capsule surrounded by lymphocytes and plasmatic cells, but in the internal core were found the remains of necrotic cells instead of macrophages, epithelioid cells, and eosinophils. No differences were observed in the inflammatory infiltrates between experimental groups.

On the other hand, lesions become more evident in the liver tissue of untreated rats, where evidence was found of granulomatous lesions. These lesions show the same organization previously described in lung; however, they were found in lesser quantity and magnitude. There was the presence of macrophages and eosinophils with a certain degree of fibromatosis (Figure 3B,E,H). Lesions are found in minor degree since it is a chronic infection and the liver is a byway organ during the migration of somatic larvae in the early stages of infection.

In kidney, an inflammatory process was observed in the renal cortex area only in the group of intact rats. Likewise, the presence of eosinophilic granulomas composed of a dense layer of collagen and core containing epithelioid cells can be noted. All other experimental groups show undamaged tissues (Figure 3C,F,I).

### Immune system’s cell subpopulation

Flow cytometry analysis was made in order to determine changes in the percentage of innate and adaptive immune cell subpopulations in the spleen, PLN and MLN of control and infected mice. Cells were first gated by size and complexity, then we selected them as T cells (CD3+); T cells were then gated as T helper (CD4+) or T cytotoxic (CD8+) (Supplementary Figure S1). B cells (CD45RA+) (Supplementary Figure S2), NK (CD161+) (Supplementary Figure S3), or Tγδ cells (TCRγδ+) (Supplementary Figure S4).

### Innate immune system’s cell subpopulation

**Figure 4:**
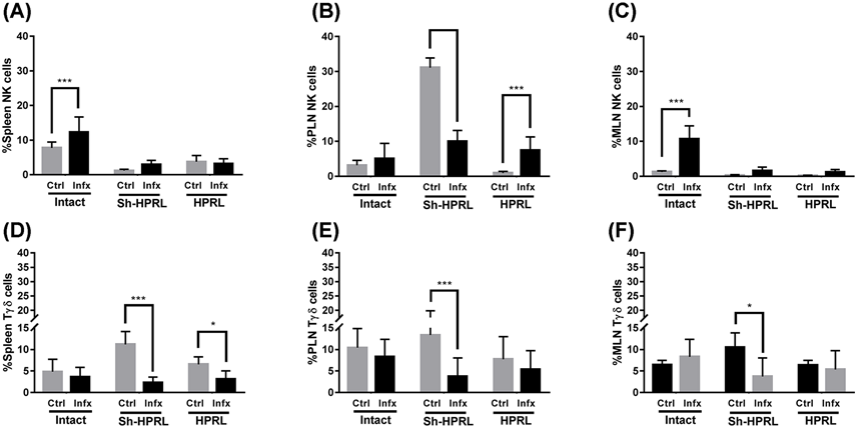
Percentage of NK and Tγδ cells in spleen, PLN and MLN, from uninfected and infected rats We used five animals per group and two rounds of experiments. Infection was performed by intragastric administration using a type Foley metallic probe at 8 weeks of age inoculating 2000 *T. canis* eggs per rat. Percentage of NK and Tγδ cells were done by Flow cytometry. Briefly, spleen, PLN and MLN were collected at the time of killing. They were disaggregated using a 70-μm sterile nylon mesh. The following primary antibodies were used: AF647 anti-rat CD161 and PE anti-rat TCRγδ. A secondary antibody solution was added with AF488 or AF647 anti-Rabbit IgG. Data analysis was performed using the software FlowJo v10.0. The data represent the mean average (± S.D.). *Significant differences due to infection status (*P<0.05, **P<0.01, ***P<0.001). Abbreviations: Ctrl, uninfected rats; HPRL, adenohypophysis implant in renal capsule surgery; Infx, infected rats; Intact, untreated rats; Sh-HPRL, simulated adenohypophysis implant in renal capsule surgery.

NK cells from the spleen may show an increase due to infection in the animals with not surgery (intact) (*P*<0.01), whereas the differences due to the surgical treatments showed a decrease in the percentage of this subpopulation in both uninfected and infected animals of the Sh-HPRL and HPRL groups (*P*<0.001) (Figure 4A). In the PLN, there is an increase in the percentage in the SH-HPRL group ctrl (*P*<0.001), whereas in the HPRL group there is an increase in the proportion due to the infection (Figure 4B). In MLN, an increase in the percentage that is due to infection in intact animals is observed (*P*<0.001), but at the moment of being submitted to the surgical procedure this difference disappears (Figure 4C). In the spleen Tγδ cells, there is an increase in the percentage of these cells in the SH-HPRL ctrl group (*P*<0.001), in addition to a decrease due to infection in the Sh-HPRL and HPRL group (*P*<0.001) (Figure 4D). In PLN and MLN, a decrease due to infection was observed in the percentage in the Sh-HPRL group (*P*<0.05) (Figure 4E,F).

### Adaptive immune system’s cells and *T. canis* specific antibody production

**Figure 5:**
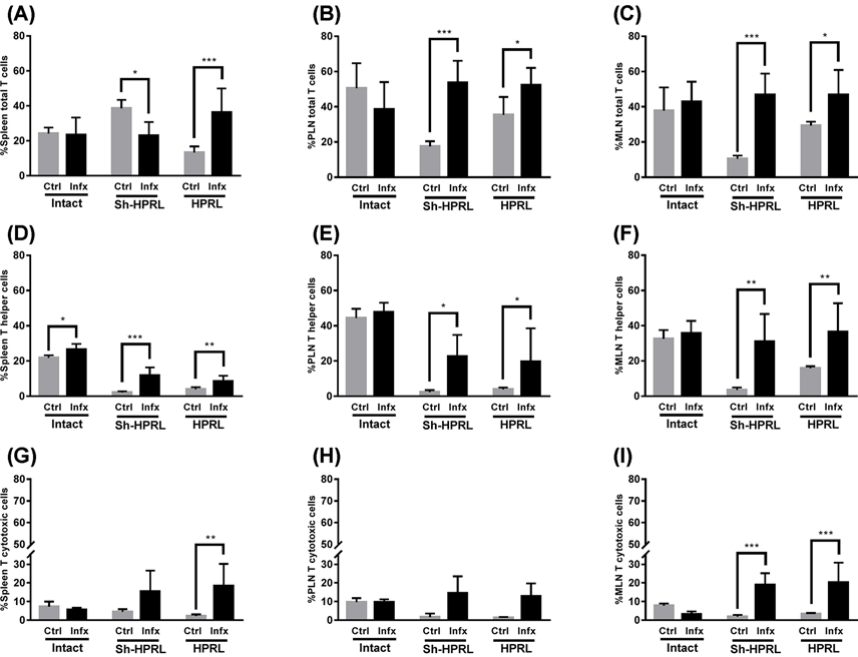
Percentage of total T cells (CD3+), Th cells (CD4+) and CTLs (CD8+) in spleen, PLN and MLN from uninfected and infected rats We used five animals per group and two rounds of experiments. Infection was performed by intragastric administration using a type Foley metallic probe at 8 weeks of age inoculating 2000 *T. canis* eggs per rat. Percentage of T cells (CD3+), Th cells (CD4+), and CTLs (CD8+) in spleen, PLN and MLN were performed by Flow cytometry. Briefly, spleen, PLN and MLN were collected at the time of killing. They were disaggregated using a 70-μm sterile nylon mesh. The following primary antibodies were used: AF 488 anti-rat CD3, PE-Cy5 Mouse anti-rat CD4, PE Mouse anti-rat CD8α, PE. A secondary antibody solution was added AF488 or AF647 anti-Rabbit IgG. Data analysis was performed using the software FlowJo v10.0. The data represent the mean average (± S.D). *Significant differences due to infection status (*P<0.05, **P<0.01, ***P<0.001). Abbreviations: Ctrl, uninfected rats; HPRL, adenohypophysis implant in renal capsule surgery; Infx, infected rats; Intact, untreated rats; Sh-HPRL, simulated adenohypophysis implant in renal capsule surgery.

The total T-cell subpopulation (CD3+) in spleen was decreased (*P*<0.001) in the uninfected HPRL group, but when infected, this decrement is compensated and the percentages become equalized compared with the remaining experimental groups (Figure 5A). In PLN, differences were observed associated with the surgical procedure in the Sh-HPRL ctrl group, in addition to increased proportions of these cells in the Sh-HPRL (*P*<0.001) and HPRL (*P*<0.05) groups caused by infection (Figure 5B). On the other hand, the MLN of the uninfected Sh-HPRL and HPRL groups are found in decreased proportion. In the latter groups, there were increased percentages due to infection (Figure 5C). Spleen Th cells showed no differences in percentage in the uninfected groups, whereas they were found decreased in the infected Sh-HPRL and HPRL groups (*P*<0.05). There were also increments (*P*<0.001) in percentage due to infection in the spleens of the uninfected Sh-HPRL and HPRL groups (Figure 5D). The PLN showed no difference (*P*>0.05) between the uninfected groups, whereas the infected Sh-HPRL and HPRL groups showed a decreased percentage of these cells; additionally, all the infected groups showed increased proportions (Figure 5E). Concerning the MLN cells, no differences were found between the infected and uninfected groups; regardless, in all cases, infection produced an increased proportion of cells (Figure 5F). Spleen CTLs showed no differences between the infected and uninfected groups (*P*>0.05) and the infection induced effect on these cells was only observed in the HPRL group, where and increased proportion was found (Figure 5G). The PLN cells showed no difference in percentage of CTLs between the treatments in both uninfected and infected groups, differences in percentage were observed only after infection in the Sh-HPRL and HPRL groups (Figure 5H). Neither there were any significant differences in MLN of the uninfected groups; on the other hand, the infected groups showed an increased percentage (*P*<0.001) of the CTL subpopulation in the Sh-HPRL and HPRL groups. Infection produced changes were present in the Sh-HPRL and HPRL groups (Figure 5I).

**Figure 6:**
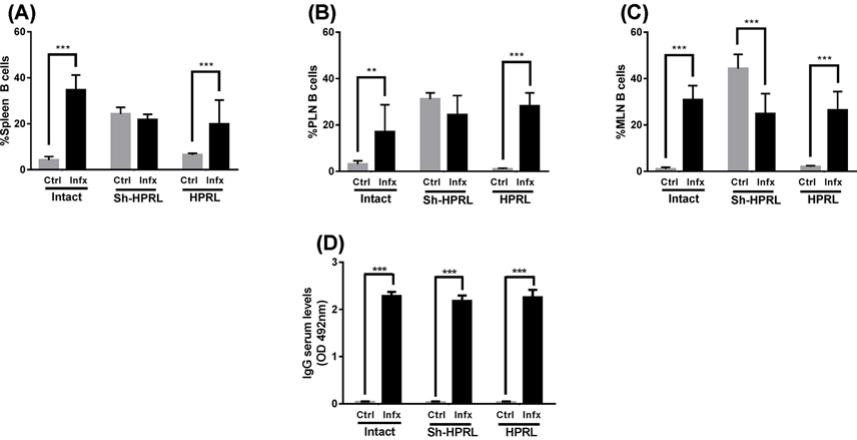
Percentage of B cells (CD45RA+) in spleen, PLN, MLN, and anti-*T. canis* antibody titration from uninfected and infected rats We used five animals per group and two rounds of experiments. Infection was performed by intragastric administration using a type Foley metallic probe at 8 weeks of age inoculating 2000 *T. canis* eggs per rat. They were performed by Flow cytometry. Briefly, spleen, PLN and MLN were collected at the time of killing. They were disaggregated using a 70-μm sterile nylon mesh. The following primary antibodies were used: PE anti-rat CD45RA. A secondary antibody solution was added AF488 or AF647 anti-Rabbit IgG. Data analysis was performed using the software FlowJo v10.0. The data represent the mean average (± S.D.). *Significant differences due to infection status (**P<0.01, ***P<0.001). TES-Ag were obtained by culturing the *T. canis* larvae according to the method described by de Savigny [17]. Purity and integrity of ESA was determined by SDS/PAGE and Coomassie staining, protein content was quantitated by Bradford method. The levels of ESA targetting antibodies in serum were measured indirectly by ELISA, Plate reading was done at 492 nm, 15 s agitation in an ELISA plate reader. The data represent the mean average (± S.D.). ***P<0.001. Abbreviations: Ctrl, uninfected rats; HPRL, adenohypophysis implant in renal capsule surgery; Infx, infected rats; Intact, untreated rats; Sh-HPRL, simulated adenohypophysis implant in renal capsule surgery.

B-cell percentages in spleen, PLN and MLN were increased due to infection in the untreated and HPRL groups, whereas in MLN a decreased percentage can be observed in the Sh-HPRL group. Increments can also be observed due to treatment in the uninfected Sh-HPRL group in three organs, and a decrement can be observed only in the spleen of infected rats in the Sh-HPRL and HPRL groups (Figure 6A–C).

Finally, parasite-specific antibodies were measured. The infection causes an increased production of IgG specific against the *T. canis* parasite (*P*<0.001) in all experimental groups, no significant differences were observed caused by the applied treatments (*P*>0.05) (Figure 6D).

## Discussion

The analysis approach for larvae reactivation was performed in three different ways: (i) by evaluating the parasitic load in different organs, particularly in the mammary gland; (ii) by determining the specific antibody titration targetting the parasite; and (iii) by observing the inflammatory process present during infection. On the other hand, the role of PRL was analyzed in different cells of the immune system, where their behavior was determined under normal, infected, and hyperprolactinemic conditions. The advantages of analyzing larvae reactivation in males are: (i) lack of estrous cycle; (ii) low PRL and female sex steroid levels; and (iii) normally undeveloped mammary glands. Although a fully developed mammary gland could not be achieved in the experimental groups, the hyperprolactinemic conditions did generate some degree of growth and, despite this PRL induced growth, no larvae migrated into these mammary glands.

Our results indicate lower larvae recuperation percentages from lungs at 70 days post-infection than those previously described by Havasiová-Reiterová et al. [19], which report a 10.23% larvae recuperation in brain at 70 days post-infection in C57BL6/J mice infected with 1000 HL. In another experiment performed on Wistar rats infected with 500 HL and killed at 60 days post-infection, only ~1% of larvae were recuperated from lungs and 3.94% from brain tissue [20]. Obtained data from gerbil infected with 1000 HL and killed at 60 days post-infection show a recuperation of 4.5% from brain, whereas no larvae where recuperated from lungs [21]. These data show that the migration of *T. canis* L2 can vary depending on factors such as the animals species utilized and time of infection, besides host type, age, and gender. Generally speaking, it could be suggested that the migration pattern in paratenic hosts is similar to that occurring in the definitive host. However, larvae distribution depends in great measure upon the species being infected, as it is likely that each one of these species possesses preferred migration sites.

In observing the inflammatory process, our results are in accord with those described by Parsons et al. (1986) [22] who reported the same type of hepatic lesions during chronic toxocariasis (8 months of infection) in BALB/c BYJ mice. Martínez reported similar lesions in lung, liver, and kidney at 60 days post-infection in gerbilis [23]. In the surgically treated groups, no lesions were found in either kidneys or liver, this could be due to an insufficient number of tissue sections performed that could have missed any inflammatory processes or that perhaps larvae had already allocated into muscle tissue, which usually contained the highest number of larvae during chronic infection. None of the experimental groups showed acute inflammatory processes that could suggest the movement of larvae into other tissues such as uterus and mammary glands.

As it has been previously mentioned, PRL has distinct effects in the modulation of the immune system, exerting paracrine and autocrine effects. As to the writing of this manuscript, there are no detailed descriptions concerning how PRL could be affecting the immune system cell subpopulations under hyperprolactinemic conditions caused by adenohypophysis transplants. Neither are there data concerning the joint effect that an additional factor could provoke, such as an antigenic challenge (in this case, the parasite *T. canis*). The findings made by the present study concerning the role of PRL over the percentages of cell subpopulations differs depending upon the type of surgery utilized, the analyzed organs (spleen, PLN, MLN) and infection status.

Concerning NK cells, it has been reported that these respond to PRL stimulation in a dose-dependent manner: PRL concentrations corresponding to a nominal plasmatic value induce cytotoxicity [24], whereas concentrations ten times higher exert a clear inhibitory effect over development, activity, and proliferation after IL-2 activation. Our results show that the percentage of NK cells was independent of plasmatic PRL concentration, although we did find increased percentages due to infection in some experimental groups. Regarding total T cells (CD3+), a decrement can be observed in the percentage of these cells in the spleen of uninfected HPRL animals. On the other hand, Th cells (CD4+) were found increased in spleen, PLN and MLN due to infection. This goes in accord with the reported increment found not only in infections by this and many other parasites [9–11]. CTLs (CD8+) presented differences due to infection in spleen, MLN and PLN in HPRL animals. The increased percentage of this subpopulation during chronic infection, in addition to the role of PRL as a Th1 cytokine, correlates with the polarization of the Th1 response present during this stage in murine models to produce IFN-γ and collaborate in the formation of eosinophilic granulomas [13]. BL (CD45RA+) are most important in the immune response against *T. canis* since they are responsible for producing specific antibodies targetting the parasite. Although there are no reports regarding BL quantity during infection, there is data showing an increased level of IgM, IgG and IgE antibodies [24]. Our results show that, indeed, the infection causes an increased percentage of BL in spleen, PLN and MLN in both untreated and HPRL groups.

Therefore, our interest was in evaluating these parameters under hyperprolactinemic conditions. No evaluation was performed on antibody levels in serum during the course of infection, performing said evaluation up until the moment of killing (70 days post-infection). In an experiment performed on gerbil, it was reported that TES-Ag targetting antibodies in serum were detected at day 10 post-infection and these levels were sustained until 130 days post-infection [21]. This increment and persistence of the antibody response is similar to that observed in humans afflicted by *visceral larva migrans* [25] due to the continuous antigenic stimuli during larvae migration and their localization in different tissues. In these results, are observed the production of specific antibodies in all the infected groups. Unlike expected, no differences were found in the remaining experimental groups. This may be due to the relatively short time of infection, which did not allow antibody level to diminish enough to be statistically different.

During toxocariasis, there is no clear evidence indicating that PRL or other hormones, are essential in the development of the infection, as it has been reported for other parasites. As previously mentioned, PRL has been broadly studied in infections caused by protozoa, where its capacity to elicit a protective Th1 response was observed, e.g. toxoplasmosis [26], leishmaniosis [27], and malaria [28], amongst others. Concerning sex steroids, it has been proved that they may have diverse effects on the advent of several parasitic diseases. Experiments performed by Larralde et al. [29] showed that 17β-estradiol favors the growth and reproduction of the parasite *Taenia crassiceps*. These results in increased parasite load in females, turning this parasitosis into a sex-linked disease. On the other hand, the effects exerted by sex steroids on the immune system become evident during gestation, impacting susceptibility, and/or resistance to parasitic infections in gestating females [30]. Along with these changes in sex steroids levels during gestation, it is important to include PRL since it is also a very important hormone during this stage. The effect of PRL and sex steroids could extend beyond an immune response into the parasite itself. In this sense, the existence is suggested of an exploiting mechanism by the parasite, enabling a faster establishment and effective reproduction, culminating in a progressive and successful infection. This transregulation phenomena have been little explored in parasites. However, there is evidence supporting the notion that a host-parasite regulation or transregulation is possible and, to date, eight parasitic species have been described in this regard including the interaction of steroid and protein hormones. It is known that the treatment *in vitro* of *Plasmodium falcipardum* merozoites with cortisol increases the number and size of gametocytes [31]. Likewise, merozoites treated with insulin, estradiol, progesterone, and testosterone considerably increased the number of gametocytes produced *in vitro*, increasing also, same as cortisol, the growth and reproduction of the parasite at this stage. The opposite occurs when these parasites are treated with 16α-bromoepiandrosterone, a dehydroepiandrosterone analog (DHEA), which induces a lower growth rate of up to 25% [32]. On the other hand and as already mentioned, DHEA treatment in cercariae, schistosomula, and adult parasites of *Schistosoma mansoni* inhibit viability and oviposition [33], whereas amastigotes of *Trypanosoma cruzi* treated with epidermal growth factor increased their proliferation [34]. With the information provided by these examples in mind, it is important to highlight the potent transregulator effect that the host’s hormones have over the parasite. Concerning *T. canis*, recently it has been demonstrated that PRL *in vitro* treatment, in larvae, accelerated their enlargement and increased their motility. Furthermore, a PRL-like receptor was identified in *T. canis* larvae, and the *in vitro* stimulation with PRL increased the number of these receptors, accelerated the growth and modified the activity of larvae [35].

Interestingly, we found and unexpected observation: PRL level raised after the infection. To our knowledge, this is the first report of PRL changes in rats infected with *T. canis*. A possible explanation is that, when infected by pathogens, including parasites, hosts display a series of responses known as acute phase reactions, which include immune, hormonal, physiological, metabolic, and behavioral changes. In a variety of host–parasite systems, hormonal changes, both in proteic and steroid hormones have been observed in the host [36]. These changes are considered to be adaptive for the parasite, since they facilitate the transmission of parasites between hosts, and/or enhance the probability that parasites are released in an appropriate location, or, the host responds to immunomodulate the response to eliminate the parasite [36]. The hormonal changes are also induced by the parasite to be able to change behaviors on the host that will help to continue life cycle. In the case of helminth infections, it has been proven that parasites can induce alterations in hormone levels [37,29]. Also, as a result of hormonal changes, the behavior of some hosts is modified, as has been observed in the mouse model infected with *T. crassiceps* [38]. Thus, it has been proposed that helminths are able to induce modifications in the hormonal levels, and behavior of their hosts, in order to facilitate their transmission. In the case of infestation with *T. canis*, it is unknown whether there is an association between the observed PRL levels and possible behavioral changes and the synthesis of antibodies, or with the particular pro-inflammatory cytokine profile induced during chronic infestation.

## Conclusion

In conclusion, the results obtained concerning PRL involvement and infection effect on the immune system differed depending upon the surgical treatment to which the rats were subjected. PRL alone may not reactivate migration of *T. canis* somatic larvae in hyperprolactinemic male rats.

## Supporting information

**supplementary Figure 1:** **Cell population analysis.** 25 Immune cell populations were defined according to the following analysis: Cells were first gated by size and complexity, then we selected them as T cells (CD3+); B cells (CD45RA+);NK (CD161+) or Tγδ cells (TCRγδ+). T cells were then gated as T helper (CD4+) or T cytotoxic (CD8+). In all cases, the percentage of PRLR+ cells was defined in histograms according to the unspecific staining in each mice of the secondary antibody used to detect the anti-PRLR.

**supplementary Figure 2:** **Immune cell 1 subpopulations comparison among experimental groups in the spleen.** Representative dot plots showing the analysis of the percentage of T helper (CD4+) vs. T cytotoxic (CD8+) cells (upper row); T cells (CD3+) vs. B cells (CD45RA+) (middle row); and NK (CD161+) vs Tγδ cells (TCRγδ+) (lower row) in the spleen of the experimental groups (from left to right): Intact Non-infected (Intact Control); Intact Infected (Intact Infx); Sham-HPRL Non-infected (Sh-HPRL Ctrl); Sham-HPRL Infected (Sh-HPRL Infx); HPRL Non-infected (HPRL Ctrl); and HPRL Infected (Sh-HPRL Infx).

**supplementary Figure 3:** **Immune cell subpopulations comparison among experimental groups in peripheral lymph nodes (PLN).** Representative dotb plots showing the analysis of the percentage of T helper (CD4+) vs. T cytotoxic (CD8+) cells (upper row); T cells (CD3+) vs. B cells (CD45RA+) (middle row); and NK (CD161+) vs. Tγδ cells (TCRγδ+) (lower row) in PLN’s of the experimental groups (from left to right): Intact Non-infected (Intact Control); Intact Infected (Intact Infx); Sham-HPRL Non-infected (Sh-HPRL Ctrl); Sham-HPRL Infected (Sh- HPRL Infx); HPRL Non-infected (HPRL Ctrl); and HPRL Infected (Sh-HPRL Infx).

**supplementary Figure 4:** **Immune cell subpopulations comparison among experimental groups in mesenteric lymph nodes (MLN).** Representative dot plots showing the analysis of the percentage of T helper (CD4+) vs. T cytotoxic (CD8+) cells (upper row); T cells (CD3+) vs. B cells (CD45RA+) (middle row); and NK (CD161+) vs. Tγδ cells (TCRγδ+) (lower row) in MLN’s of the experimental groups (from left to right): Intact N 49 on-infected (Intact Control); Intact Infected (Intact Infx); Sham-HPRL Non-infected (Sh-HPRL Ctrl); Sham-HPRL Infected (Sh-HPRL Infx); HPRL Non-infected 1 (HPRL Ctrl); and HPRL Infected (Sh-HPRL Infx).

## Abbreviations

BL: B lymphocyte
CTL: cytotoxic T lymphocyte
DHEA: dehydroepiandrosterone analog
EE: embryonated egg
IL: interleukin
MLN: mesenteric lymphatic node
PLN: peripheral lymph node
PRL: prolactin
PSS: physiological saline solution
TES-Ag: targetting excretion and secretion antigen
Th: helper T lymphocyte
TL: T lymphocyte
UNAM: Universidad Nacional Autónoma de Mexico
