# Supplementary material for: Prolactin as immune cell regulator in Toxocara canis somatic larvae chronic infection
Source: Biosci Rep. 2018 Jul 31;38(4):BSR20180305. doi: 10.1042/BSR20180305 (PMC6066655; doi:10.1042/BSR20180305)
Supplement: Supplementary file 1 [file bsr20180305_Supp1.pdf]

SUPPLEMENTARY MATERIAL

Prolactin as immune cell regulator in *Toxocara canis* somatic larvae chronic infection.

Víctor Hugo Del Río-Araiza[a], Karen Elizabeth Nava-Castro[b], Fernando Alba-Hurtado[c], Andrés Quintanar-Stephano[d], Hugo Aguilar-Díaz[e], Marco Antonio Muñoz-Guzmán[c], Pedro Ostoa-Saloma[a], María Dolores Ponce-Regalado[f] and Jorge Morales-Montor[a*]

[a] Departamento de Inmunología, Instituto de Investigaciones Biomédicas, Universidad Nacional Autónoma de México, AP 70228, México D.F. 04510, México.

[b] Departamento de Genotoxicología, Centro de Ciencias de la Atmósfera, Universidad Nacional Autónoma de Mèxico.

[c] Departamento de Ciencias Biológicas, Facultad de Estudios Superiores Cuautitlán, Universidad Nacional Autónoma de México, México.

[d] Centro de Ciencias Básicas, Universidad Autónoma de Aguascalientes. Aguascalientes, México.

[e] Centro Nacional de Investigación Disciplinaria en Parasitología Veterinaria, Instituto Nacional de Investigaciones Forestales Agrícolas y Pecuarias, INIFAP. Jiutepec, Morelos, México.

[f] Universidad de Guadalajara. Centro Universitario de los Altos - Departamento de clínicas. Carretera a Yahualica, Km. 7.5. Tepatitlán de Morelos, Jalisco, México.

Jorge Morales-Montor *Corresponding author: Tel: +5255 56232673; E-mail address: jmontor66@biomedicas.unam.mx, jmontor66@hotmail.com

Supplementary Figure 1. Cell population analysis. Immune cell populations were defined according to the following analysis: Cells were first gated by size and complexity, then we selected them as T cells (CD3+); B cells (CD45RA+);NK (CD161+) or Tγδ cells (TCRγδ+). T cells were then gated as T helper (CD4+) or T cytotoxic (CD8+). In all cases, the percentage of PRLR+ cells was defined in histograms according to the unspecific staining in each mice of the secondary antibody used to detect the anti-PRLR.

Supplementary Figure 2. Immune cell 1 **subpopulations comparison among experimental groups in the spleen.** Representative dot plots showing the analysis of the percentage of T helper (CD4+) vs. T cytotoxic (CD8+) cells (upper row); T cells (CD3+) vs. B cells (CD45RA+) (middle row); and NK (CD161+) vs Tγδ cells (TCRγδ+) (lower row) in the spleen of the experimental groups (from left to right): Intact Non-infected (Intact Control); Intact Infected (Intact Infx); Sham-HPRL Non-infected (Sh-HPRL Ctrl); Sham-HPRL Infected (Sh-HPRL Infx); HPRL Non-infected (HPRL Ctrl); and HPRL Infected (Sh-HPRL Infx).

Supplementary Figure 3. Immune cell subpopulations comparison among experimental groups in peripheral lymph nodes (PLN). Representative dotb plots showing the analysis of the percentage of T helper (CD4+) vs. T cytotoxic (CD8+) cells (upper row); T cells (CD3+) vs. B cells (CD45RA+) (middle row); and NK (CD161+) vs. Tγδ cells (TCRγδ+) (lower row) in PLN's of the experimental groups (from left to right): Intact Non-infected (Intact Control); Intact Infected (Intact Infx); Sham-HPRL Non-infected (Sh-HPRL Ctrl); Sham-HPRL Infected (Sh-HPRL Infx); HPRL Non-infected (HPRL Ctrl); and HPRL Infected (Sh-HPRL Infx).

Supplementary Figure 4. Immune cell subpopulations comparison among experimental groups in mesenteric lymph nodes (MLN). Representative dot plots showing the analysis of the percentage of T helper (CD4+) vs. T cytotoxic (CD8+) cells (upper row); T cells (CD3+) vs. B cells (CD45RA+) (middle row); and NK (CD161+) vs. Tγδ cells (TCRγδ+) (lower row) in

MLN’s of the experimental groups (from left to right): Intact Non-infected (Intact Control); Intact Infected (Intact Infx); Sham-HPRL Non-infected (Sh-HPRL Ctrl); Sham-HPRL Infected (Sh-HPRL Infx); HPRL Non-infected 1 (HPRL Ctrl); and HPRL Infected (Sh-HPRL Infx).

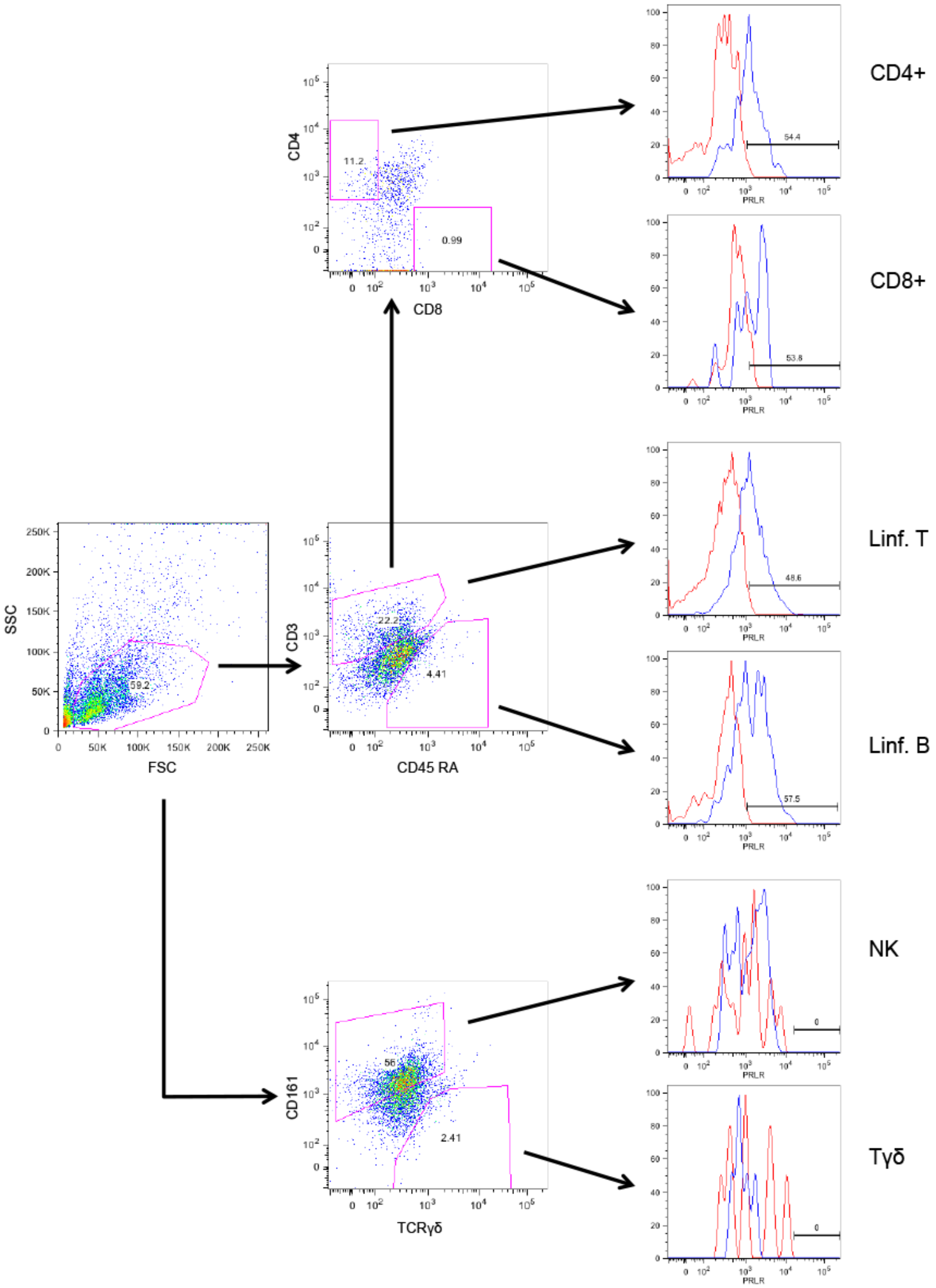

SSC
FSC
59.2
CD3
CD45 RA
22.2
4.41
CD4
CD8
11.2
0.99
CD161
TCRγδ
56
2.41
PRLR
54.4
53.8
48.6
57.5
0
0
CD4+
CD8+
Linf. T
Linf. B
NK
Tγδ

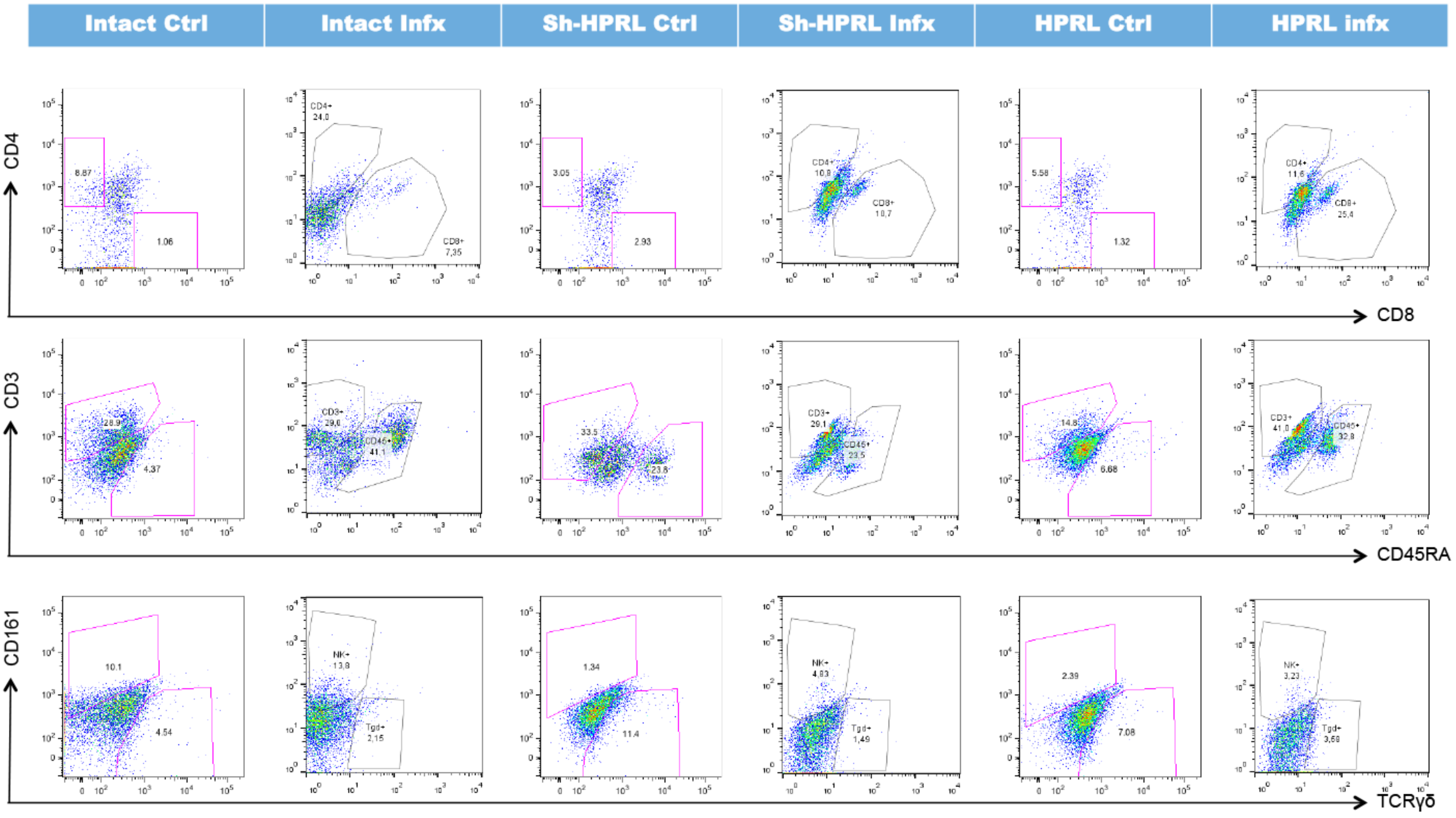

Intact Ctrl
Intact Infx
Sh-HPRL Ctrl
Sh-HPRL Infx
HPRL Ctrl
HPRL infx
CD4
CD8
CD3
CD45RA
CD161
TCRγδ

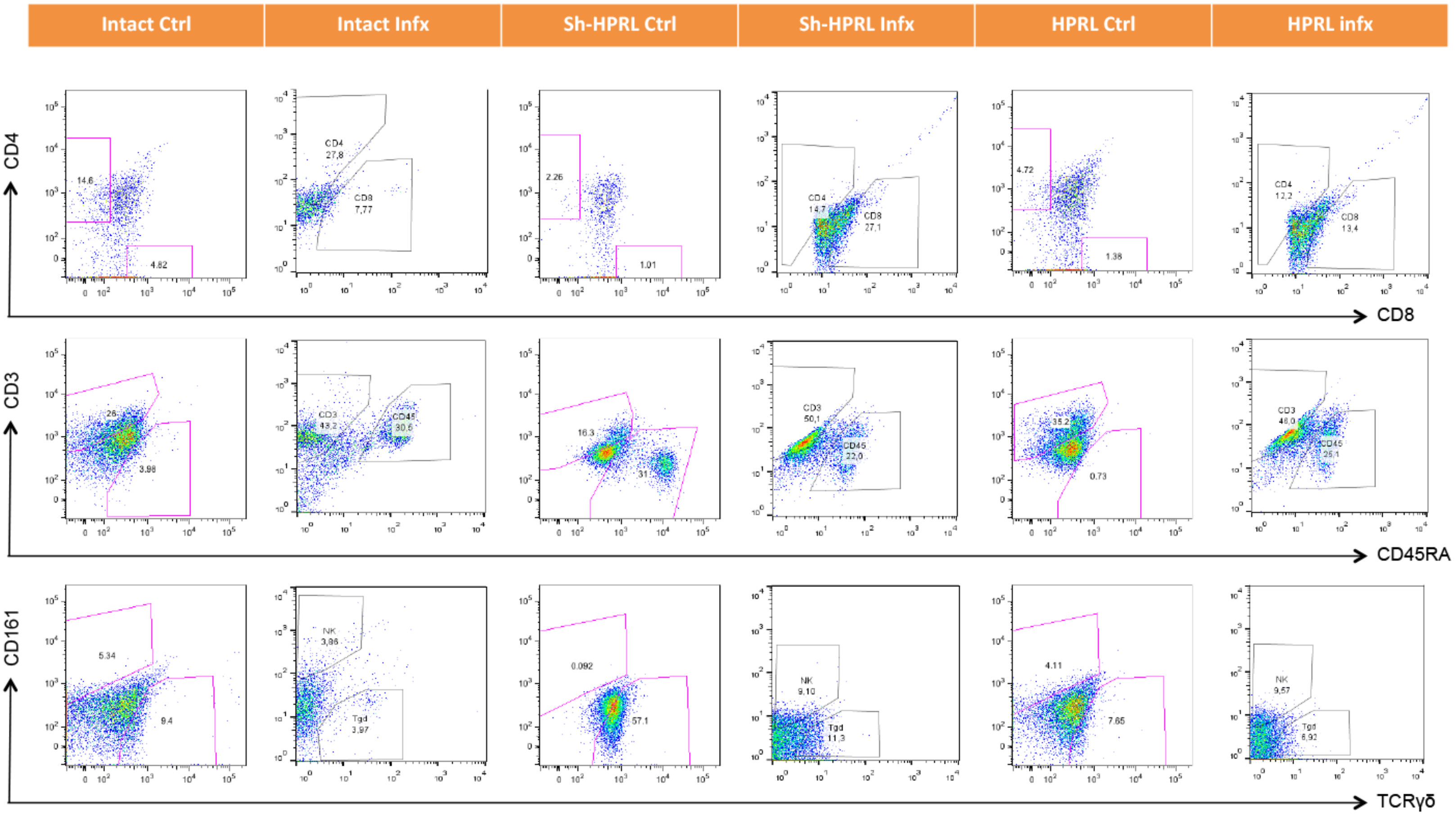

Intact Ctrl
Intact Infx
Sh-HPRL Ctrl
Sh-HPRL Infx
HPRL Ctrl
HPRL infx
CD4
CD8
CD3
CD45RA
CD161
TCRγδ

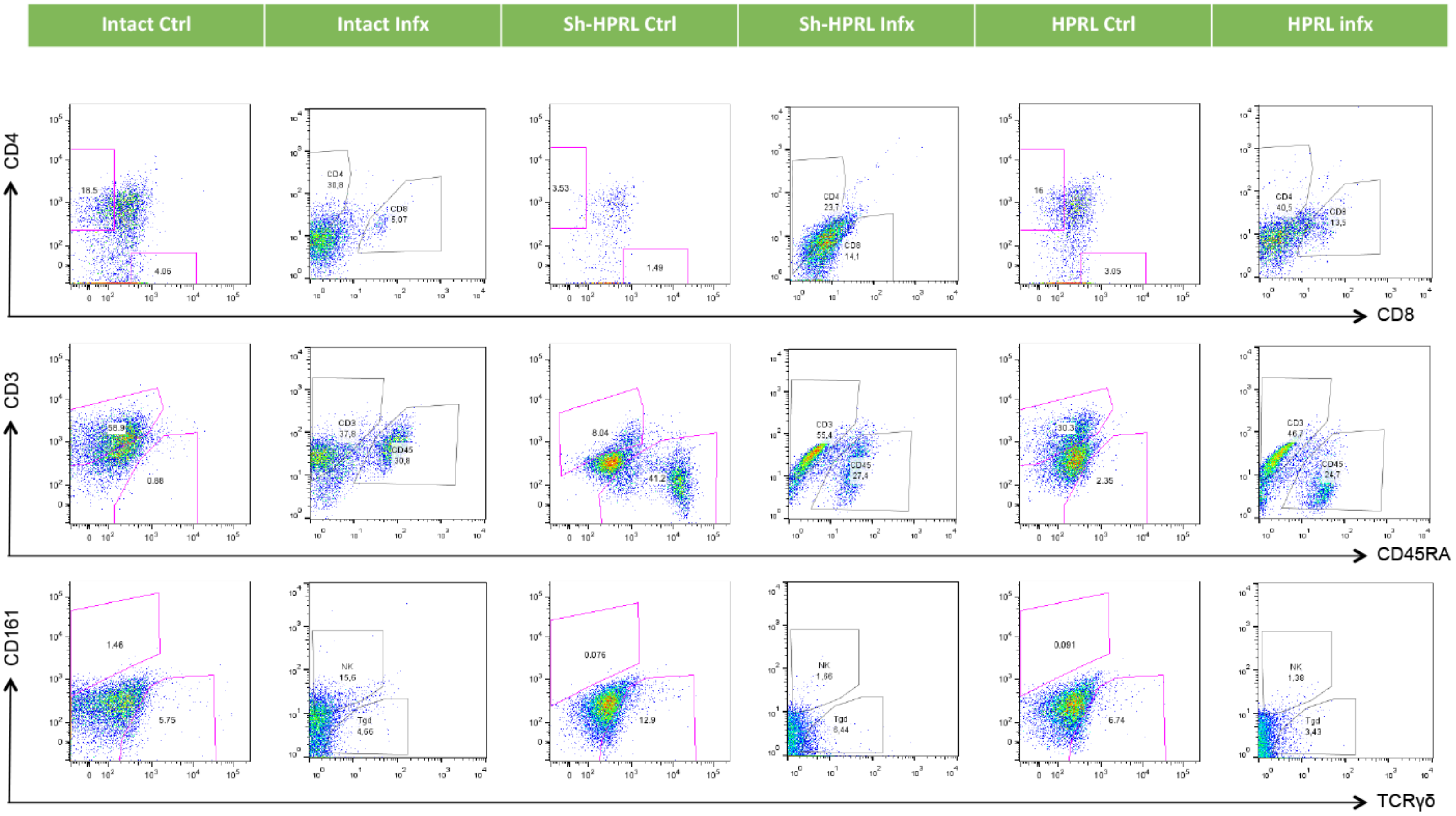
Intact Ctrl
Intact Infx
Sh-HPRL Ctrl
Sh-HPRL Infx
HPRL Ctrl
HPRL infx
CD4
CD8
CD3
CD45RA
CD161
TCRγδ
18.5
4.06
CD4
30,8
CD8
5,07
3.53
1.49
CD4
23,7
CD8
14,1
16
3.05
CD4
40,5
CD8
13,5
58.9
0.88
CD3
37,8
CD45
30,8
8.04
41.2
CD3
55,4
CD45
27,4
30.3
2.35
CD3
46,7
CD45
24,7
1.46
5.75
NK
15,6
Tgd
4,66
0.076
12.9
NK
1,86
Tgd
6,44
0.091
6.74
NK
1,38
Tgd
3,43
